# Incorporating Information From Electronic and Social Media Into Psychiatric and Psychotherapeutic Patient Care: Survey Among Clinicians

**DOI:** 10.2196/13218

**Published:** 2019-07-12

**Authors:** Katherine W Hobbs, Patrick J Monette, Praise Owoyemi, Courtney Beard, Scott L Rauch, Kerry J Ressler, Ipsit V Vahia

**Affiliations:** 1 McLean Hospital Belmont, MA United States; 2 Harvard Medical School Cambridge, MA United States

**Keywords:** technology, social media, psychotherapy, psychiatry

## Abstract

**Background:**

Obtaining collateral information from a patient is an essential component of providing effective psychiatric and psychotherapeutic care. Research indicates that patients’ social and electronic media contains information relevant to their psychotherapy and clinical care. However, it remains unclear to what degree this content is being actively utilized by clinicians as a part of diagnosis or therapy. Moreover, clinicians’ attitudes around this practice have not been well characterized.

**Objective:**

This survey aimed to establish the current attitudes and behaviors of outpatient clinicians regarding the incorporation of patients’ social and electronic media into psychotherapy.

**Methods:**

A Web-based survey was sent to outpatient psychotherapists associated with McLean Hospital in Belmont, Massachusetts. The survey asked clinicians to indicate to what extent and with which patients they reviewed patients’ social and electronic media content as part of their clinical practice, as well as their reasons for or against doing so.

**Results:**

Of the total 115 respondents, 71 (61.7%) indicated that they had viewed at least one patient’s social or electronic media as part of psychotherapy, and 65 of those 71 (92%) endorsed being able to provide more effective treatment as a result of this information. The use of either short message service text messages or email was significantly greater than the use of other electronic media platforms (χ^2^_1_=24.1, n=115, *P*<.001). Moreover, the analysis of survey responses found patterns of use associated with clinicians’ years of experience and patient demographics, including age and primary diagnosis.

**Conclusions:**

The incorporation of patients’ social and electronic media into therapy is currently common practice among clinicians at a large psychiatric teaching hospital. The results of this survey have informed further questions about whether reviewing patient’s media impacts the quality and efficacy of clinical care.

## Introduction

Electronic and social media platforms have become ubiquitous and essential tools in navigating the 21st century [1]. Content stored and shared on these platforms, therefore, contains extensive information about our everyday lives and interactions [2]. Research in the mental health field has begun to explore whether electronic and social media content may contain clinically relevant markers of individuals’ behavior and mental health. During this period of rapid uptake in utilization of these platforms, it is important to understand how they may reflect, impact, or be used to augment treatment for mental health conditions. In this study, we use the term electronic and social media platforms to refer to apps that individuals access on their mobile phones, tablets, or computers for the purpose of communicating with or sharing content with others. The most prominent examples of these platforms include Facebook, Instagram, email, short message service (SMS) text messaging, and other messaging apps.

A growing body of literature has begun to examine how we may leverage electronic media usage to identify markers of psychiatric illness or response to treatment. Studies analyzing electronic media content have identified differences in language use between healthy individuals and individuals with different psychiatric disorders, suggesting that clinically relevant signals across a wide variety of illnesses can be found in the language used on social media platforms [3,4]. Likewise, a recent study found that analysis of language in Facebook posts can predict the existence of a depression diagnosis in medical records with a similar degree of accuracy as established depression screening surveys [5]. Another study noted that patients’ word usage in emails was a predictor of therapeutic outcomes in a sample of individuals with social anxiety [6]. Another study correlated the frequency of social media posting with health outcomes—those who posted most frequently on Facebook in their sample were more likely to have a diagnosis or positively screen for depression [7].

In examining social media postings, researchers have developed computational models to better predict onsets of psychiatric illnesses. One such study analyzed the Twitter activity of users who reported a clinical depression diagnosis [8]. Researchers retroactively examined participants’ Twitter content from the year before the onset of depression to measure markers of social activity, emotions, and relational concerns. They utilized these markers to create a model predicting the likelihood of depression onset before a clinical diagnosis [8]. In a similar study, researchers created computational methods to screen for markers of depression and posttraumatic stress disorder (PTSD) in Twitter users’ posts [9]. They reviewed the Twitter data of individuals with depression and PTSD from the year before their clinical diagnosis and those of healthy subjects. They found their computational models could distinguish content from healthy and depressed subjects and demonstrated improved accuracy in correctly diagnosing depression and PTSD as compared with the general performance of practitioners [9].

Simultaneously, research has begun to explore patient attitudes toward sharing these electronic media data in the context of clinical care. A recent study surveyed new patients in a psychiatric outpatient setting regarding their use of mobile phones and their willingness to share data collected on their phones with mental health providers [10]. Overall, the survey found moderate willingness among patients in a psychiatric outpatient setting to share data collected via a mobile phone with their clinicians and, unsurprisingly, respondents were more likely to grant a mental health app access to less personal content [10]. Research has also explored mental health providers’ general attitudes toward gathering information about their patients through electronic sources but has not explicitly addressed how clinicians are utilizing patients’ own social media content to inform care [11]. For example, in one study, 20% of responding psychiatrists and psychologists reported searching online for information about their patients either *sometimes* or *often* [11]. Of these clinicians, 45% said they would perform a search to cross-check information, 35% would do so out of curiosity, and 60% would do so to gather information. The study did not specify what type of information clinicians were gathering, nor how this information impacted the care they delivered [11].

Studies such as these indicate that electronic and social media can be leveraged as effective predictors and trackers of mental illness, but it remains unclear to what degree this type of content is being actively utilized by clinicians as a part of diagnosis or therapy. Thus, gaps remain regarding how mental health clinicians are utilizing patients’ electronic and social media to identify clinically relevant information to improve care and how they are weighing potential benefits and drawbacks of doing so. To address these gaps, we surveyed mental health clinicians who provide outpatient psychotherapy. The goal of the survey was to establish to what degree clinicians are currently accessing patients’ social and electronic media content, how they are doing so, in what ways they are incorporating this content into therapy, and what concerns they have around this practice. Notably, our project did not seek to characterize electronic communication between clinicians and patients for the purpose of scheduling, follow-up, and other clinical needs.

## Methods

### Respondents

The survey population consisted of 115 outpatient psychotherapy clinicians associated with McLean Hospital in Belmont, Massachusetts, who completed a Web-based survey regarding their use of social and electronic media as part of their standard clinical care. The principal investigator emailed the survey to the 244 individuals included on the McLean Hospital master outpatient clinician mailing list. Emails were sent out in 2 rounds of email blasts between April 19, 2018, and June 6, 2018. The first round of emails went to 102 outpatient clinicians who work within formal hospital clinics, and the second went to 142 clinicians doing hospital-based private practice. For both rounds, reminder emails were sent 3, 5, and 8 days following the original email. Of the 244 email recipients, 13 email addresses were deemed undeliverable, resulting in 231 valid email addresses. With 115 total respondents out of 231 recipients, the overall response rate for this survey was 49.8%. The respondents spanned a range of professional disciplines and reflected the diversity of psychotherapy provided at McLean hospital. The email link to the survey specified that the purpose of the survey was to gain an understanding of whether and how clinicians were accessing electronic media in regular care. We did not collect demographic information on clinicians.

### Survey Instrument

We were unable to identify an existing instrument that would adequately allow us to capture the breadth of information regarding clinician attitudes and behaviors around use of electronic media. Hence, we developed our own instrument designed by consensus among senior study investigators (KR, CB, and IV) to capture naturalistic use of electronic media in therapy. We built in items to facilitate stratification by qualification and years of experience. We elected to limit information about clinician demographics to minimize survey time and maximize response rate. We next built items to capture what types of electronic media platforms were used and in which patient populations this approach had been tried. Finally, we developed items to capture with greater detail the process of accessing this information, maintaining privacy, and its usefulness.

The survey, which is available in Multimedia Appendix 1, was created using Google Forms and consisted of 17 questions. First, respondents were asked to indicate their professional degree and number of years of clinical experience. Respondents were then asked to respond yes or no to whether they had viewed a patient’s social or electronic media as part of outpatient psychotherapy. If they responded no, they were asked whether or not they had considered viewing this content and the major factors or concerns they might consider in making the decision.

If they responded yes, they were prompted to answer 12 more questions aimed at characterizing their use of this content in psychotherapy. Clinicians were asked which platforms they accessed and how they accessed them—whether it was directly with the patient in the session, outside of the session with the patient’s permission, or indirectly via report from the patient or their loved ones. The survey also asked with which age demographic and clinical populations the clinicians used this approach, with how many patients they used it, and in approximately how many sessions per patient. Following these questions, clinicians were asked whether they believed access to this information helped improve the quality of care they delivered. Additional details were collected around the process of accessing this information—namely, which party had suggested access to this content and whether they had discussed issues around privacy. Finally, 2 free-response questions allowed respondents to share their reasons for and concerns about accessing this type of content in their clinical care.

### Data Safety and Storage

The survey was distributed via a private Google Survey invitation to clinicians’ professional secure email addresses. Only those with the link were able to access it. Data from the survey were downloaded and saved on private Partners Healthcare servers. No identifying information was collected, unless the respondents chose to volunteer such information at the end of the survey; there was space for respondents to give their name and email address if they were interested in learning more about technology-related projects being conducted at the hospital. This question was removed from the dataset before data analysis to deidentify the responses.

### Data Analysis

All data analyses were performed using the SPSS 3 (IBM SPSS Statistics) statistical package. For quantitative items, descriptive statistics were generated, and then respondents were stratified by years of clinical experience and by professional degree, followed by a comparison of differences using chi-square tests. For questions in which respondents had the option to write free-text responses, these responses were consolidated into categories upon consensus by study staff and then recoded accordingly. As the volume of qualitative data was relatively low and we had distinct statements from each respondent, we elected not to use customized qualitative analysis software. Instead, we adapted a simple qualitative classification approach [12] and sorted qualitative statements into broad themes which this study investigators agreed to by consensus.

This project was undertaken as a quality improvement initiative at McLean Hospital and as such was not formally supervised by the institutional review board per their policies. The institutional review board provides a checklist [13] to determine whether a specific project qualifies as a quality improvement initiative. A total of 4 of the study investigators (KH, PM, PO, and IVV) assessed this study simultaneously and independently using this checklist, and all determined that the project met criteria to qualify as a QII. We also communicated this to the institutional review board who confirmed that if the study meant QII, formal supervision was not required. We utilized the existing mechanism for mass email communication with McLean clinicians to appropriately disseminate the survey, and we specified that responding to the survey was voluntary and part of an information gathering process.

## Results

### Respondent Demographics

Of the 115 clinicians who responded to the survey, 31 (27.0%) hold MDs as their professional degree, 35 (30.4%) hold PhDs, and 30 (26.1%) hold Licensed Clinical Social Worker (LCSW) degrees. Other degrees represented in smaller numbers include: Advanced Practice Registered Nursing degrees, MD/PhD, Licensed Mental Health Counselor degrees, and MA degrees. Moreover, 47 (40.9%) respondents have less than 10 years of clinical experience, 29 (25.2%) have 10 to 20 years of experience, and 39 (33.9%) have more than 20 years of experience.

### Overall Viewing Rates

Of the 115 respondents, 71 (61.7%) indicated that they had viewed at least one patient’s social or electronic media as part of psychotherapy. The remaining 44 (38.3%) indicated that they had never viewed a patient’s electronic media. A total of 1 respondent who indicated having viewed a patient’s media did not answer all the subsequent questions regarding this content. Therefore, some of the follow-up questions for clinicians who reported yes to viewing media had 71 total responses and some had only 70 responses.

Of the 44 respondents who have not viewed patient’s media, 10 (23%) said that they have considered incorporating this content into therapy sessions.

### Types of Media Viewed

Table 1 summarizes the breakdown of patients’ social and electronic media platforms that clinicians viewed. A total of 60 of 70 respondents to this question (86%) viewed SMS text messages and 56 (80%) viewed emails, making these the most frequently accessed platforms. The use of either SMS text messages or email was significantly greater than the use of any of the other electronic media platforms (χ^2^_1_=24.1, N=115, *P*<.001).

**Table 1:** Type of media viewed by clinicians.

Media type	Responses (yes), n (%)
SMS (short service message)	60 (86)
Email	56 (80)
Facebook	27 (39)
Call history	13 (19)
Instagram	12 (17)
Blogs	7 (10)
Twitter	7 (10)
Snapchat	6 (9)
WhatsApp	5 (7)

### Clinical Experience and Media Use

A summary of media viewing according to respondents’ clinical experience is shown in Table 2. A total of 33 of the 47 respondents (70%) with less than 10 years of experience and 20 of the 29 respondents (69%) with 10 to 20 years of experience stated they had viewed a patient's social or electronic media; meanwhile, 18 of the 39 respondents (46%) with more than 20 years of experience reported having done so. The relationship between years of experience and viewing rates was significant (χ^2^_2_=6.1, N=115, *P*=.048). We also performed similar analyses stratifying clinicians by their professional degree but did not note any significant differences between groups or even nonstatistically significant trends.

### Methods of Viewing Media

Of the 70 respondents to this question who have viewed patients’ electronic or social media, 62 (89%) reported having viewed this content directly in the patients’ presence, and 46 (66%) indicated having gathered information about the media content through patient self-report, as demonstrated in Table 3.

**Table 2:** Overall clinician utilization of patient electronic media information in psychotherapy.

Media type^a^	Total yes responses, n (%)	<10 years clinical experience, n (%)	10-20 years clinical experience, n (%)	20+ years clinical experience, n (%)	Chi-square (df)	*P* value (2-sided)
Email (n=70)	56 (80)	23 (33)	17 (24)	16 (23)	2.5 (2)	.28
Text (n=70)	60 (86)	28 (40)	17 (24)	15 (21)	0.2 (2)	.92
Facebook (n=70)	27 (39)	14 (20)	8 (11)	5 (7)	1.3 (2)	.53
Instagram(n=70)	12 (17)	5 (7)	4 (6)	3 (4)	0.2 (2)	.92
Call history (n=70)	13 (19)	4 (6)	3 (4)	6 (9)	3.5 (2)	.17
Whatsapp (n=70)	5 (7)	1 (1)	3 (4)	1 (1)	2.7 (2)	.26
Twitter (n=70)	7 (10)	4 (6)	1 (1)	2 (3)	0.8 (2)	.67
Blogs (n=70)	7 (10)	0 (0)	3 (4)	4 (6)	7.1 (2)	.03
Snapchat (n=70)	6 (9)	2 (3)	1 (1)	3 (4)	2.1 (2)	.36
Any	70 (100)	32 (46)	20 (29)	18 (25)	5.4 (2)	.07

^a^We used chi-square tests to compare clinicians who responded *yes* with using a specific media platform by years of clinical experience.

**Table 3:** Clinician's methods of accessing patients' electronic or social media content.

Method of access	Responses (yes), n (%)
Viewed content directly in patient's presence	62 (89)
Patient self-report	46 (66)
Outside of session with permission	14 (20)
Report from friends or relatives	12 (17)

### Patient Demographic

Clinicians reported viewing social or electronic media with the following patient age groups: 20 of 70 (29%) adolescents, 46 (66%) young adults, 46 (66%) adults, and 5 (7%) older adults.

Moreover, clinicians indicated accessing this content most commonly with patients diagnosed with depression or anxiety; 42 of 70 (60%) of yes respondents stated that they viewed media with patients diagnosed with either or both conditions, followed by 32 (46%) with borderline personality disorder. Each of the remaining diagnoses yielded less than 35% of yes responses, as indicated in Tables 4 and 5.

### Viewing Frequency

A total of 9 of 70 (13%) clinicians who viewed their patient’s electronic or social media reported doing so only 1 time, whereas 40 (57%) clinicians reported doing so very infrequently. A total of 18 of 70 (26%) reported viewing media content in roughly every 5 to 10 sessions per patient, and 2 (3%) reported viewing it every 2 to 3 sessions. Finally, 1 (1%) reported viewing this content in every session.

Moreover, 12 of 70 (17%) clinicians who viewed their patient’s electronic or social media reported doing so with just 1 to 2 patients, 26 (37%) reported doing so with 3 to 5 patients, 14 (20%) reported doing so with 6 to 10 patients, and 18 (26%) reported doing so with more than 10 patients.

### Whose Idea?

A total of 52 of 70 (74%) clinicians reported that it was the patient’s idea to incorporate social and electronic media into psychotherapy, whereas 10 (14%) clinicians reported that it was their own idea. A total of 7 (10%) indicated that it was a mutual suggestion, 1 (1%) reported that it was a family member’s idea, and 1 (1%) indicated that multiple parties suggested it.

### Impact on Treatment

We incorporated a single item asking clinicians to rate the extent to which they were able to provide more effective treatment in part because of accessing their patients’ electronic or social media. A total of 17 of 70 (24%) clinicians reported noting *significant* improvement in the level of care, whereas 30 (42%) reported seeing *moderate* improvement, 18 (25%) reported *slight* improvement, and 6 (8%) reported *no improvement* in their ability to deliver effective care. There were no differences on this measure when we stratified clinicians by years of experience or highest level of training.

### Privacy

We inquired whether clinicians had discussed privacy with patients and, if so, who initiated the conversation. A total of 44 of 71 (62%) clinicians who had accessed patients’ social and electronic media indicated that they discussed issues of privacy with the patients. We found that clinicians with greater experience were significantly more likely (χ^2^_4_=13.2, N=115, *P*=.01) to bring up privacy concerns with their patients; 16 of the 18 (89%) clinicians with more than 20 years of experience who viewed this content reported having a conversation around privacy, whereas 12 of 20 (60%) of those with between 10 and 20 years of experience and 16 of 33 (48%) of those with less than 10 years of experience who viewed this content reported doing so.

The majority of clinicians who discussed privacy initiated the conversations themselves; only 2 of 71 (3%) clinicians reported that their patients raised privacy concerns regarding sharing this type of content.

### Reasons for Accessing This Content

Of the 71 respondents who indicated having accessed this content, 63 (89%) provided free-text explanations of their motivations for accessing patients’ media as part of clinical care. Using a qualitative analytic approach described in the Methods section, we categorized those responses by consensus into the following 5 general thematic categories, listed below in order of frequency. If comments fell into multiple categories, they were counted under each relevant category. Therefore, the percentages add to 117, rather than 100.

1. To monitor or address a specific target behavior (26/63, 41%)
2. To provide feedback on patients’ electronic communications and behaviors (26/63, 41%)
3. To obtain collateral information on patients’ life (15/63, 24%)
4. To establish working alliance/rapport (4/63, 6%)
5. Logistical reasons (3/63, 5%)

### Concerns About Accessing This Content

Of the 71 respondents who indicated having accessed this content, 48 (68%) provided free-text explanations of their concerns regarding accessing this content. On the basis of our qualitative analytic approach described in the Methods section, responses fell into 7 general thematic categories, listed below in order of frequency. In this case, all responses fell into just 1 category, and thus, were counted once.

1. Privacy concerns/ethical boundary (15/48, 31%)
2. No concerns as long as done on patients’ own terms (10/48, 21%)
3. Detracts or distracts from therapy (7/48, 15%)
4. Content is subjective and easily misinterpreted (6/48, 13%)
5. None (6/48, 13%)
6. Time constraint (2/48, 4%)
7. Other (2/48, 4%)

**Table 4:** Media viewing by patient age demographic

Patient age demographic	Responses (yes), n (%)
Adults	46 (66)
Young adults	46 (66)
Adolescents	20 (29)
Older adults	5 (7)

**Table 5:** Media viewing by patient diagnosis.

Patient diagnosis	Responses (yes), n (%)
Anxiety	42 (60)
Depression	42 (60)
Borderline personality disorder	32 (46)
Posttraumatic stress disorder	22 (31)
Bipolar disorder	21 (30)
Psychotic disorders	17 (24)
Obsessive compulsive disorder	42 (60)
Eating disorders	7 (10)

## Discussion

### Conclusions

This survey addresses the naturalistic use of patients’ electronic and social media by mental health clinicians. With a 49.8% (115/231) response rate, the survey results provide useful insights into the current practices among a diverse group of therapists. We noted that the majority of outpatient clinicians surveyed (115/71, 61.7%) reported having viewed at least one patient’s electronic or social media as part of care, with email and SMS text messaging emerging as the most frequently accessed platforms by far. We also found that clinicians who had been in practice for fewer years accessed this information more frequently than more experienced clinicians, recognizing that this is confounded by age cohort of the clinicians as well. This may reflect a greater proficiency among more junior clinicians in understanding how technology may be intrinsic to daily life [14,15]. Conversely, this may also reflect a lesser-perceived need for concrete collateral information among more experienced therapists, who may practice with a better-established frame [16].

Another prominent finding was that only 3% of clinicians who reported viewing social or electronic media indicated that any of their patients had voiced concerns around privacy. This was a surprisingly low number, which may reflect patient confidence in allowing therapists to access this information, or a hesitancy to disagree with a therapist’s suggestion because of power dynamics. It may also be related to our finding that the majority of clinicians accessed these data during sessions, in the patients’ presence. Accessing such personal information face to-face and giving patients control over the ability to share this information may have helped foster a sense of the privacy of the session extending to electronic data as well [17]. The ability to access collateral information, whether from family members [18] or through patient writings or other forms of expressive therapy [19], has long been a tradition in psychotherapy. Accessing electronic communications represents an extension of this tradition, incorporating contemporary means of communication and leveraging technology that may enable extraction on deeper behavioral signals from natural language.

Overall, our finding that a high percentage of clinicians are accessing electronic media and doing so with patients present—and in over 74% of cases, at patients’ suggestion—indicates that the process of incorporating this information into therapy is a common and organic process across various provider types (MD, PhD, LCSW). Clinicians reported accessing more private data, such as email and SMS text messaging, to a greater extent than social media, which may indicate clinicians’ perceptions that these more personal platforms contain more relevant behavioral signals. Our finding that 92% of clinicians reported that accessing electronic or social media improved their ability to provide effective treatment points to a role for increased attention to electronic media in care. Therefore, there is an imperative to explore whether this perception of benefit holds true in observational and experimental studies, which quantify potential benefit in a controlled manner and to understand the mechanisms underlying any observed benefit. Future studies are also needed to establish best practice standards that guide the appropriate access and use of this type of content across different providers.

### Limitations

Our findings should be interpreted within the context of the limitations of this survey. Although our respondents spanned a range of degrees and years of experience, we only surveyed clinicians affiliated with a single psychiatric institution in the Boston region, which may impact the generalizability of the findings. Furthermore, the patient population served by McLean Hospital therapists may not be representative of the population at large, which in turn may impact the representativeness of the sample with regard to use of electronic media as well.

In addition, our survey was designed to be brief, anonymous, and easy to complete to maximize response rates, rather than to be comprehensive. Consequently, we did not obtain details on provider demographics. We also did not ask clinicians to share the breakdown of diagnoses or age groups that they see in their outpatient practices, so results regarding media viewing by these variables may be skewed by the makeup of the overall patient population seen by clinicians in our sample. Collection of these data would be important in follow-up work. Although we do not believe this is a common occurrence, we did not specifically inquire whether clinicians accessed patients’ data without patients’ permission.

We acknowledge that our survey did not address all of the ways in which clinicians may be utilizing electronic platforms to obtain clinical information about a patient’s wellbeing. Specifically, we did not ask about the use of apps to track patients’ mood and behavior over time, for example, through daily mood or activity logs on one’s mobile phone. Apps of this type are emerging within psychotherapy, and clinicians may utilize these behavioral tracking methods in addition to or in lieu of the informal methods of accessing and discussing patients’ social and electronic media content that we laid out in the survey.

Finally, limitations associated with surveys, including responder bias and recall bias, apply to this work as well. As we did not track provider demographics, and the survey was anonymized, we are not able to compare responders with nonresponders and draw insights into the effects of responder bias. We recommend that future work, including from our team, study this issue to gain a better understanding of predictors of using electronic media in therapy.

Despite these limitations, our work provides an early indication that therapists in practice are incorporating information from digital platforms into the care process. Although evidence regarding the impact of this information is limited, our findings identified 2 primary purposes for accessing the information—to monitor and address a specific target behavior and to provide feedback on patients’ electronic communications and behaviors. This finding suggests that the additional information serves as a valuable tool in the therapeutic process beyond just collecting collateral information.

### Future Directions

Future work on this topic should focus on replicating our initial findings in larger, more diverse clinician populations. More data are also needed to determine, in a more specific manner, exactly how and when electronic media may enhance or hinder the therapy process. Issues around privacy and confidentiality of this information also merit thoughtful discussion. This, in turn, may help develop a more systematic approach toward optimally utilizing electronic and social media in the augmentation of the therapeutic process in mental health care settings.

## Appendix

Multimedia Appendix 1 Text of the survey that was sent to clinicians via a Google Form.

## Abbreviations

LCSW: Licensed Clinical Social Worker
PTSD: posttraumatic stress disorder
SMS: short message service
