# Supplementary material for: Incorporating Information From Electronic and Social Media Into Psychiatric and Psychotherapeutic Patient Care: Survey Among Clinicians
Source: J Med Internet Res. 2019 Jul 12;21(7):e13218. doi: 10.2196/13218 (PMC6659389; doi:10.2196/13218)
Supplement: Multimedia Appendix 1 [file jmir_v21i7e13218_app1.pdf]

Appendix 1. Social and Electronic Media Use in Psychotherapy Survey

A team of McLean researchers led by Drs. Ipsit Vahia and Kerry Ressler are initiating a study aimed at assessing whether reviewing patients' electronic communication data (email, text messages, social media) may impact the process of therapy. In developing the study protocols, we are seeking feedback from McLean clinicians to help understand current trends in utilizing electronic media for therapy, and to help design the process for reviewing electronic media.

This survey will be used to better understand how clinicians are accessing and utilizing patients' social and electronic media to inform their treatment plans and therapy sessions. The research team will use this information to inform the study design.

For clinicians who are not currently using this type of data, we would like to hear your perspectives on this type of work. This may include foreseen benefits and drawbacks, potential strategies for implementation and use in treatment, or any other general feedback on the concept.

This survey will be completely anonymous, and we will not be tracking responders. If you would like to speak to the researchers or participate in future focus groups on this topic, you may provide contact information at the end of the survey.

<u>Section 1</u>

What is your highest professional degree? (select one)

- MD
- PhD
- LMHC
- LCSW
- LPS
- Other...

How many years of clinical experience do you have? (select one)

- Less than 5 years
- 5-10 years
- 10-15 years
- 15-20 years
- 20-25 years
- 25-30 years
- 30+ years

Have you ever viewed a patient's social or electronic media as part of a therapy session? This may include accounts such as Facebook or Twitter, but also emails or texts. (select one)

- Yes (→ continue to section 2, then proceed to section 4)
- No (→ go to section 3)

<u>Section 2</u>

What type of media have you viewed as part of care? (select all that apply)

- Facebook
- Twitter
- Email
- Text
- Call history
- Personal Blogs
- Snapchat
- Instagram
- WhatsApp
- Other…

How have you accessed or viewed this information? (select all that apply)

- Self-report from patient
- Direct viewing of media with patient present
- Outside of visit with patient's permission
- Information shared from relatives/caregivers
- Other…

With what age demographic of patients are you viewing this media with? (select all that apply)

- Adolescents
- Young Adults
- General Adult Population
- Older Adults

About how many patients have you accessed this content with? (select one)

- 1-2
- 3-5
- 6-10
- 10+

In about how many sessions per patient do you access this content? (select one)

- Only once
- Very infrequently
- Every 5-10 sessions
- Every 2-3 sessions
- Every session

Whose idea was it to use this information in your sessions? (select one)

- Clinician
- Patient
- Other…

With what patient population do you use this with? (select all that apply)

- Depression
- Anxiety
- Bipolar
- PTSD
- OCD
- BPD
- Schizophrenia
- Schizoaffective
- Eating Disorders
- Memory-related
- Other…

Do you think you're able to provide more effective treatment in part due to the use of this type of information? (select one)

- Not at all
- Slightly
- Moderately
- Significantly
- Other…

Have you had a conversation about the privacy of the content? (select one)

- Yes
- No

Have your patients raised concerns about privacy? (select one)

- Yes
- No

What are your concerns about using this type of information? (free response)

Why have you chosen to view this information? What purpose does it serve for you as a clinician? (free response)

<u>Section 3</u>

Have you ever considered using electronic/social media to inform your therapy sessions? (select one)

- Yes
- No

In considering whether to use patient's electronic communications/social media in the context of therapy, what are major factors or concerns for you?

- Usefulness
- Takes additional time

- Worried about impact on rapport
- Patient privacy
- N/A
- Other…

Section 4

Would you be interested in joining a focus group of clinicians on this topic?

- Yes
- No
- Maybe

Would you like more information on a planned study assessing whether/how access to patients' electronic or social media impacts therapy?

- Yes
- No
- Maybe

If you are interested in learning more about the research or speaking with the research team directly please provide your name and contact information below. (free response)
